# Selection of treatment for hepatic epithelioid hemangioendothelioma: a single-center experience

**DOI:** 10.1186/s12957-019-1729-y

**Published:** 2019-11-07

**Authors:** Linping Cao, Jiawei Hong, Lingfeng Zhou, Yufu Ye, Yuanxing Liu, Jun Yu, Shusen Zheng

**Affiliations:** 1 Division of Hepatobiliary and Pancreatic Surgery, Department of Surgery, First Affiliated Hospital, School of Medicine, Zhejiang University, Hangzhou, 310003 China; 2 Key Laboratory of Combined Multi-Organ Transplantation, Ministry of Public Health, First Affiliated Hospital, School of Medicine, Zhejiang University, Hangzhou, 310003 China

**Keywords:** Hepatic epithelioid hemangioendothelioma, Surgery, Transplantation, Radiofrequency ablation

## Abstract

**Background:**

Hepatic epithelioid hemangioendothelioma (HEHE) is a rare angiogenic tumor with no recognized effective treatment. Treatment options used worldwide include liver transplantation (LT), liver resection (LR), radiofrequency ablation (RFA), chemotherapy, and observation. The aim of this study was to describe the efficacy of different treatment options used for HEHE at our center.

**Methods:**

The medical charts of 12 patients with HEHE (9 women and 3 men) who were diagnosed and treated at the First Affiliated Hospital of Zhejiang University, China, between January 2011 and December 2017 were retrospectively reviewed.

**Results:**

The patients were diagnosed by postoperative histopathology or fine needle aspiration biopsy. Two patients with diffuse lesions received LT and were alive without recurrence at the last follow-up. Three patients received LR as the initial treatment, and all of them developed recurrence during the follow-up period. One patient received RFA and remained free of disease, while the remaining six patients opted for simple observation rather than treatment. One of the patients who received LR passed away because of tumor recurrence within 32 months after surgery; the other patients showed no significant disease activity after treatments for their recurrent lesions. As of April 2018, the mean follow-up duration was 39.6 ± 20.1 months (15–82 months).

**Conclusions:**

There are multiple strategies for HEHE. Considering its indolent course, initial observation for assessment of the lesion behavior may aid in the selection of appropriate treatment. Surgery or LT is suitable for patients with disease progression during the observation period. However, our sample size was small, and further studies are required to gather more information that can aid in optimal treatment selection.

## Background

Epithelioid hemangioendothelioma (EHE) is a rare malignant tumor with a vascular origin, and it primarily occurs in the liver. The incidence rate for hepatic EHE (HEHE) is approximately 1–2 per million individuals, while the mortality rate is between 40 and 65% [1, 2]. HEHE is recognized as a low-grade tumor with a clinical course that lies intermediate to that of haemangioma and that of angiosarcoma. Both liver lobes are involved in most patients, although the right lobe is more susceptible than the left one, regardless of the presence of single or multiple lesions. Some patients also exhibit extrahepatic disease (EHD) at the time of diagnosis, with involved sites including the lungs, bones, and regional lymph nodes, among others [1]. Histopathological examination is required for definitive diagnosis. Currently, there is no known effective treatment, with liver transplantation (LT), liver resection (LR), radiofrequency ablation (RFA), chemotherapy, and observation being reported in studies around the world [3]. The aim of the present study was to describe the efficacy of different treatment options used for HEHE at our center.

## Patients and methods

This retrospective study included 12 patients diagnosed with HEHE on the basis of postoperative histopathology or fine needle puncture and treated between January 2011 and December 2017 at our hospital. The medical records of all patients were retrospectively reviewed for the following data: demographic characteristics, results of physical examinations and blood tests, treatments, findings of histopathology, and disease-free/overall survival.

Patients who opted for observation without treatment were recalled at 1 month after diagnosis. Subsequently, they were evaluated every 3 months until the last follow-up (April 2018). Patients who received treatment were also evaluated every 3 months after treatment completion. Regular physical examinations, blood tests, and imaging examinations were performed at every visit. The final status of patients was categorized as living with disease, living without disease, and death. Disease-free survival was calculated from the time of diagnosis to the time of recurrence, while overall survival was calculated from the time of diagnosis to the time of death or the last follow-up, whichever was earlier.

All participants in this research were diagnosed as HEHE at the First Affiliated Hospital, Zhejiang University School of Medicine, Zhejiang, China. The research was approved by the Ethical Review Committee of this hospital. And informed consent was obtained from the study participants according to the guidelines of the Declaration of Helsinki.

## Results

A total of 12 patients diagnosed with and treated for HEHE were enrolled in this study. The basic clinical characteristics of all patients are presented in Table 1. The mean age of patients was 47.9 ± 11.8 years (range 27–70 years). Six patients received treatment (one man and four women), while the remaining six (two men and four women) opted for observation without any treatment after diagnosis via biopsy. Among the six treated patients, two (patients 5 and 12) with diffuse, extensive hepatic lesions received LT. One of these patients underwent the procedure twice because of biliary complications after the first procedure. Another three patients received LR as their initial treatment, while the remaining one received RFA. Another six patients chose follow-up without any treatments after diagnosis.

**Table 1:** Clinical data of hepatic epithelioid hemangioendothelioma patients

Patient	Age	Gender	Follow-up duration	Number of tumor	Metastasis	Pathology			Treatment	Recurrence	Death
						F8-R-Ag	CD31	CD34			
1	44	M	82	3	/	(+)	(+)	(+)	Observation	/	/
2	56	F	32	> 5	/	(+)	(+)	(+)	LR	(+)	(+)
3	27	F	63	3	/	(+)	(+)	(+)	RFA	/	/
4	40	F	53	3	Lung	(+)	(+)	(+)	Observation	/	/
5	34	F	52	> 5	/	(+)	(+)	(+)	LTs	/	/
6	52	F	47	3	/	(+)	(+)	(+)	Observation	/	/
7	43	F	36	> 5	/	(+)	(+)	(+)	LR + RFA	(+)	/
8	43	F	28	4	/	(+)	(+)	(+)	LR + RFA + chemotherapy	(+)	/
9	50	F	25	4	/	(+)	(+)	(+)	Observation	/	/
10	70	F	21	> 5	/	(+)	(+)	(+)	Observation	/	/
11	60	M	21	> 5	Lung	(+)	(+)	(+)	Observation	/	/
12	56	M	15	> 5	Lymph nodes	(+)	(+)	(+)	LT	/	/

*LT* liver transplantation, *LR* liver resection, *RFA* radiofrequency ablation

The two patients who received LT did not exhibit significant tumor progression during the follow-up periods of 52 and 15 months, respectively. Neither of them received adjuvant therapy after LT. One of the patients who received LR (patient 2) passed away because of tumor recurrence within 32 months after the surgery. Another patient (patient 7) who received LR for multiple lesions in the right lobe received RFA for local recurrence at 2 months after the surgery. No obvious disease activity was observed in the lesion area after RFA. The third patient (patient 8) who received LR exhibited positive margins in histopathological examination of surgical specimens. Seven courses of docetaxel-based chemotherapy were administered; however, enhanced magnetic resonance imaging (MRI) exhibited tumor recurrence at 18 months after the surgery. RFA was performed, and the patient survived without recurrence until the last follow-up. The patient (patient 3) who received RFA exhibited three lesions in the right lobe; the largest mass measured 3.5 × 3.0 cm. Contrast-enhanced computed tomography (CT) performed after ablation showed no obvious enhancement in the lesion areas, and the patient remained free of disease during the follow-up period.

None of the six patients who opted for observation exhibited significant tumor progression at the last follow-up visit, and they continued to receive examinations and surveillance CT at 3-monthly intervals. For one survivor, positron emission tomography performed at 82 months revealed a suspected metastasis in the superior lobe of the right lung. Another patient who presented with diffuse liver involvement and exhibited suspected lung metastasis survived for 21 months and was alive at the time of writing this manuscript. Although LT or chemotherapy with sorafenib was recommended for this patient, it was not accepted.

## Discussion

**Fig. 1:**
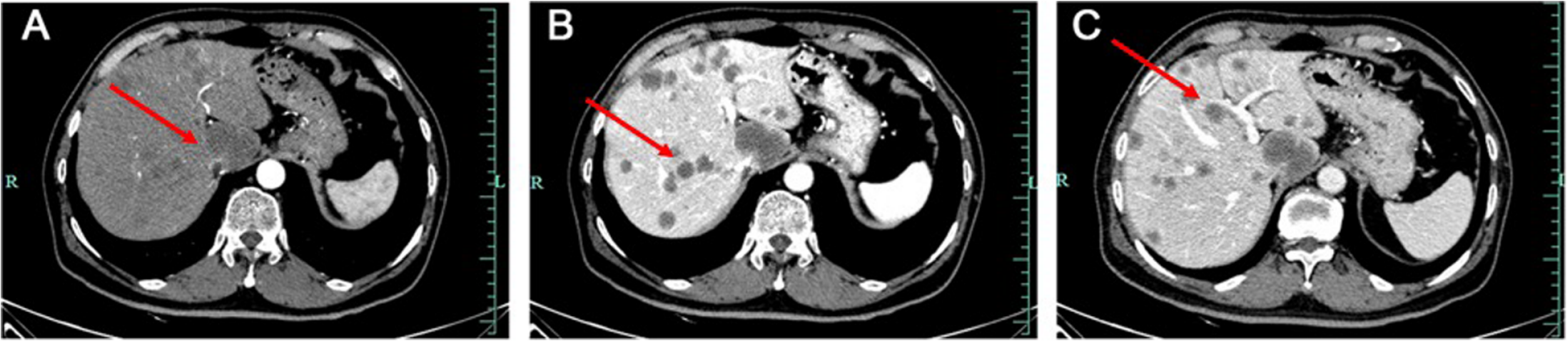
Abdominal enhanced computerized tomography (CT) images of a hepatic epithelioid hemangioendothelioma patient (arrows). **a** Multiple low-density nodular lesions scattered in the liver parenchyma involving the right lobe and left medial segment in arterial phase. **b**, **c** Multiple low-density lesions with inhomogeneous enhancement in venous phase (**b**) and delayed phase (**c**)

**Fig. 2:**
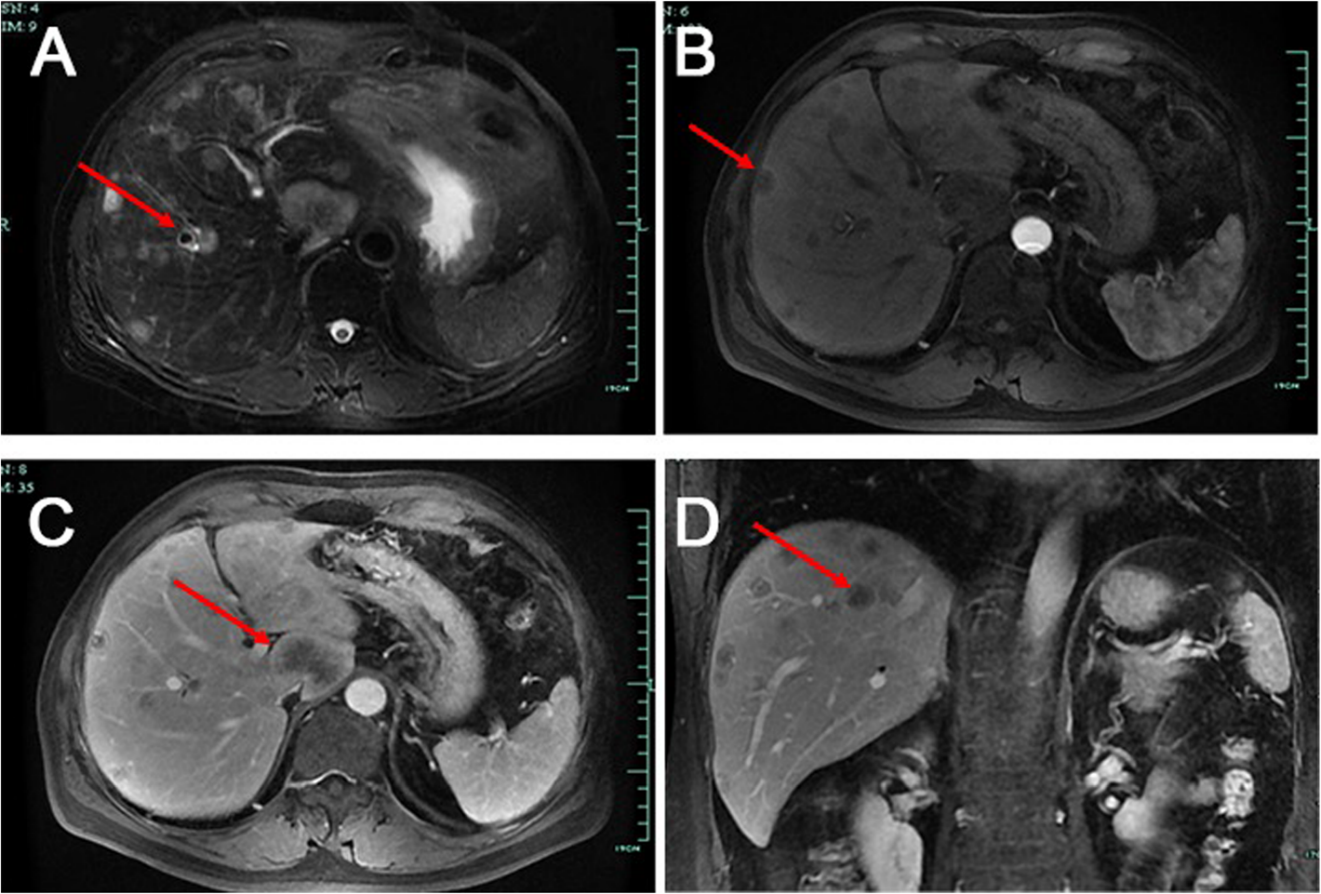
MRI scan images of a hepatic epithelioid hemangioendothelioma patient (arrows). **a** T2-weighted MRI showing multiple lesions with low signal. **b** Artery venous phase showing multiple lesions with hyperintensity. **c** Portal venous phase showing multiple lesions with a heterogeneous mild to moderate hyperintensity. **d** Coronal view image showing multiple lesions in the liver

Ishak et al. first reported HEHE [4]. HEHE exhibits a variety of clinical manifestations that range from the absence of symptoms to portal hypertension. In the present study, more than half the patients visited our hospital with nonspecific hepatic lesions identified by physical examination. Multiple hepatic masses were frequently observed in imaging examinations such as ultrasound, CT, and MRI, which are used for HEHE diagnosis [5]. CT can adequately detect hepatic lesions, which mostly exhibit low density [6]. Seventy-six percent of the patients had homogeneous enhancement on enhanced CT scan [5, 7]. CT scan can detect the area around the poor blood flow lesion which is often rich in blood flow that causes the shrink of Glisson’s capsule [8]; this is one of the clues for HEHE diagnosis. In the present study, all patients exhibited masses with low density on CT images, although the enhancement features were not consistent, which is a diagnostic limitation (Fig. 1). In MRI, most lesions exhibited low signal intensity with a peripheral dark rim on T1-weighted images and high signal intensity on T2- weighted images (Fig. 2) [1]. Gadoxetic acid-enhanced MRI and diffusion-weighted MRI may also play significant roles in the differentiation of HEHE from hemangioma and angiosarcoma [9, 10].

**Fig. 3:**
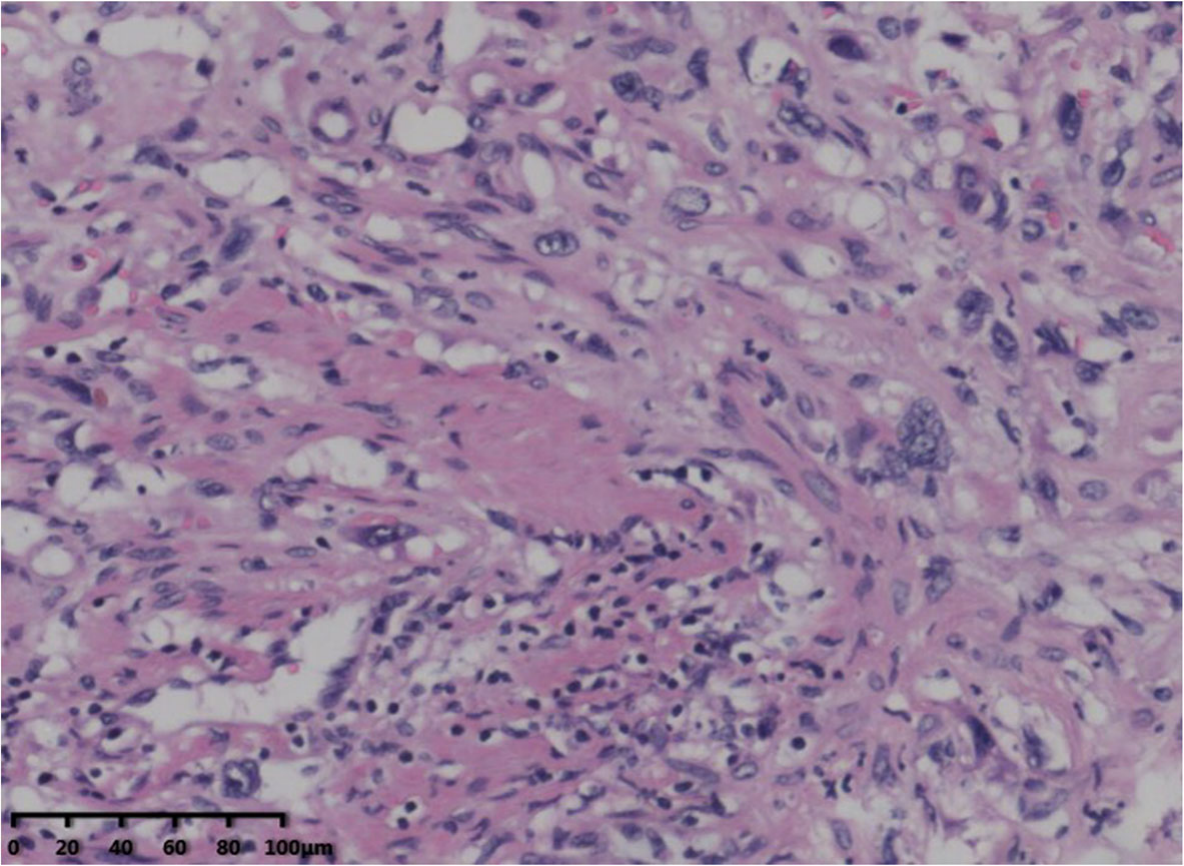
Hematoxylin-eosin staining showed abnormal hyperplasia of fibrous tissue combined with vessel-like formations, with scattered neoplastic epithelioid cells (× 200)

**Fig. 4:**
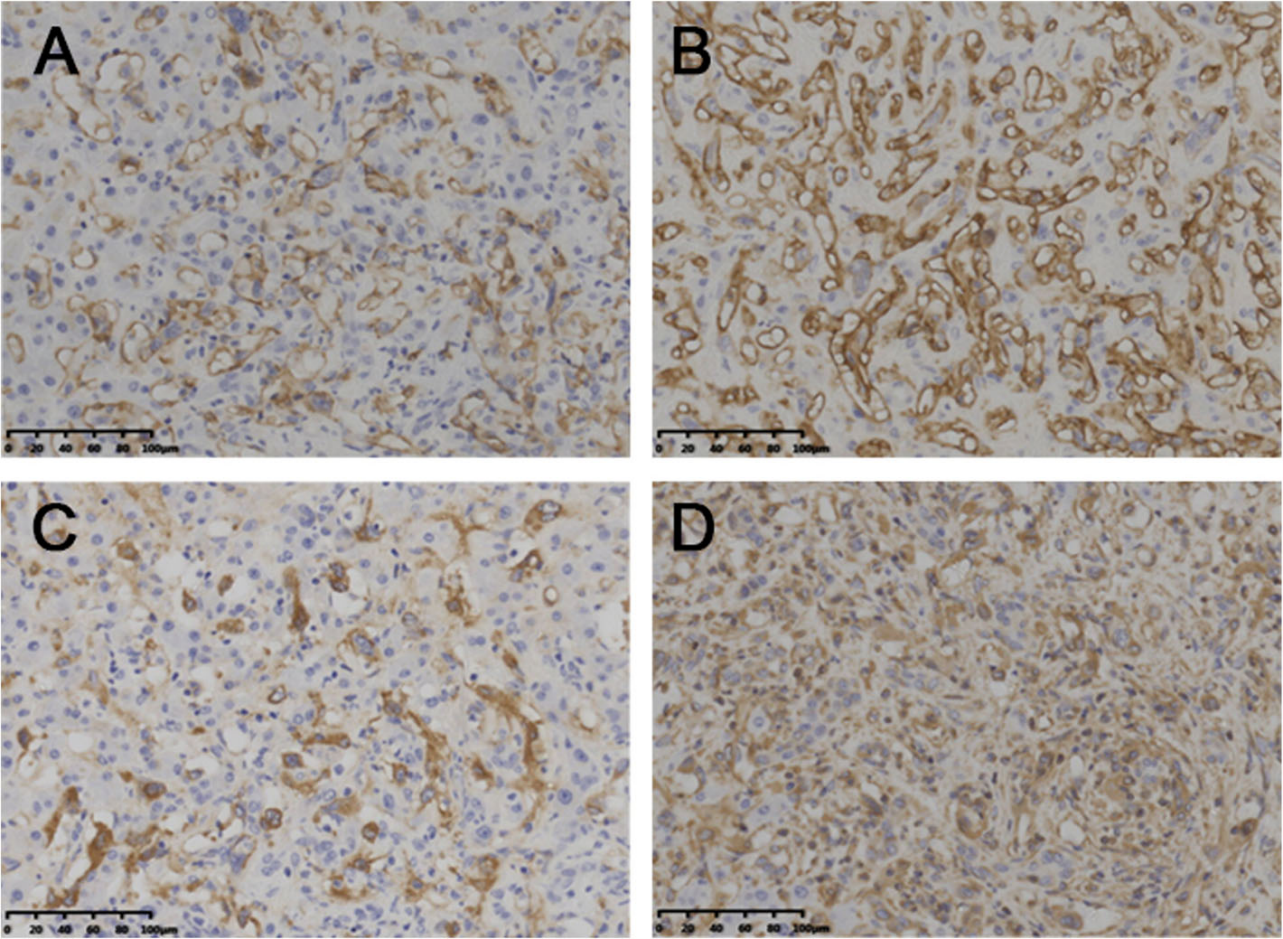
Pathological investigations identified hepatic epithelioid hemangioendothelioma. **a** Immunohistochemistry showed that the tumor was positive for CD31 (× 200). **b** Immunohistochemistry showed that the tumor was positive for CD34 (× 200). **c** Immunohistochemistry showed that the tumor was positive for F8-R-Ag (× 200). **d** Immunohistochemistry showed that the tumor was positive for Vimentin (× 200)

Diagnosis of HEHE primarily depends on immunohistochemical evidence of endothelial differentiation and the findings of histopathological examination, because the clinical and biological characteristics of this lesion are similar to those of hemangioma and angiosarcoma [11]. HEHE displays an infiltrative growth pattern, with epithelioid, dendritic, and intermediate cells interspersed in a matrix rich in hyaluronic acid (Fig. 3) [1, 12]. Immunohistochemical analysis can reveal positivity for angiogenic markers such as Factor VIII-related antigen, CD31, and CD34 and negative for cytokeratins [13, 14]. The reported rates of positivity for Factor VIII-related antigen, CD31, and CD34 are 100%, 86%, and 94%, respectively [1]. In the present study, all patients exhibited positivity for all three markers (Fig. 4).

The treatment of HEHE must take into account the involved liver lobes, the number and size of lesions, and EHD. However, there is no single effective treatment because of the rarity of the tumor and the consequent lack of a consensus regarding the optimal treatment worldwide. Mehrabi et al. reported that LT is the most common treatment, followed by observation without treatment, chemotherapy or radiotherapy, and liver resection [1].

LT is suitable for patients with multiple unresectable lesions, regardless of the presence or absence of EHD [15, 16]. The 5-year survival rate after LT is 50–89% [15]. In a European study involving 149 patients who underwent LT [6], the mean survival duration was 7.6 years (2.8–14.4 years), and risk factors for recurrence included macrovascular invasion and hilar lymph node invasion. The 1-, 3-, and 5-year survival rates were 97.1%, 88.6%, and 74.4%, respectively. Another European study involving 59 patients demonstrated that vascular invasion, rather than lymph node invasion or EHD, is a risk factor for a poor prognosis after LT for HEHE [15]. In general, LT has been proposed as the treatment of choice because of the hepatic multicentricity, of HEHE, as observed in the present study.

LR has been the treatment of choice for patients with resectable lesions in a single lobe or lesions exhibiting feasibility for complete R0 resection [17]. A previous study showed that < 10 lesions, a tumor diameter of < 10 cm, and < 4 extrahepatic lesions were favorable factors for LR [18]. Another study mentioned that LR should be strictly limited to patients with single lobe involvement [19]. Mehrabi et al. [20] also reported three patients who developed recurrence after LR and survived for > 100 months. However, the recurrence rate after LR is higher than that after LT. In the present study, the prognosis after LR was poorer than that after LT in patients with lesions larger than 10 cm. Meanwhile, RFA or chemotherapy can be considered as adjuvant therapy for recurrence after LR.

With regard to chemotherapy, a study from The Royal Marsden Hospital reported that the median survival duration after chemotherapy can reach 9.8 years and that combined treatment with interferon-alpha and 5-fluorouracil can achieve favorable effects [18]. In addition, the use of immunosuppressants or mammalian target of rapamycin inhibitors could benefit patients [21]. In a phase II clinical trial [22], sorafenib chemotherapy could extend the survival duration by > 6 months in approximately 33.5% patients. However, some scholars reported that HEHE is insensitive to chemotherapy or exhibits a poorer response to chemotherapy than to LR [23, 24]. Even in the present study, docetaxel treatment showed no obvious effects, and the patient eventually developed recurrence. This indicates that HEHE may not be sensitive to first-line chemotherapy drugs. Another patient with suspected metastasis who refused sorafenib therapy survived for > 15 months without obvious disease progression; this shows the unpredictable nature of HEHE.

**Fig. 5:**
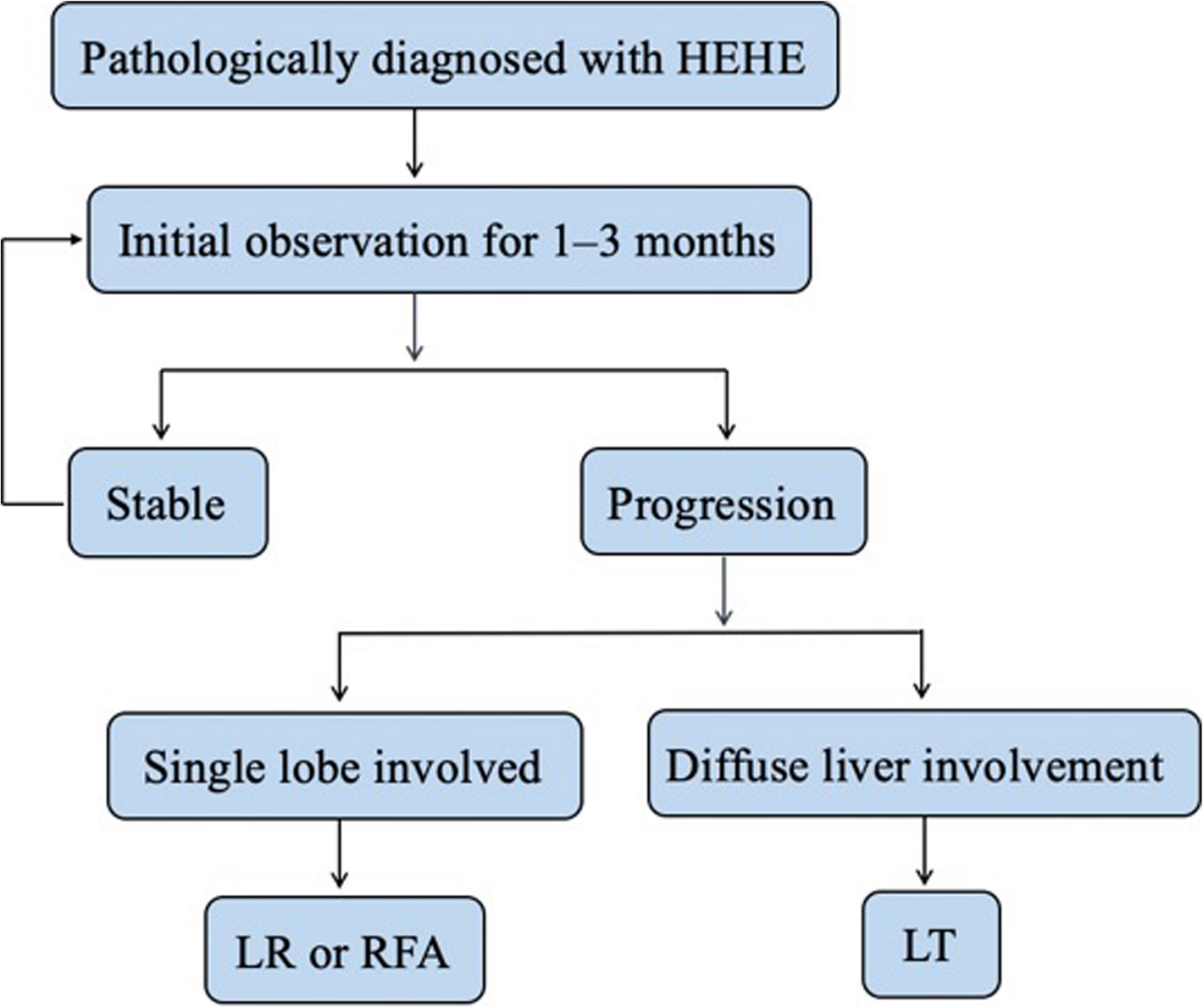
Flow diagram of the initial treatment regimen for the patients diagnosed with hepatic epithelial haemangioendothelioma (HEHE). LR, liver resection; RFA, radiofrequency ablation; LT, liver transplantation

The clinical course of patients who are simply observed after HEHE diagnosis remains unpredictable. Makhlouf et al. [2] reported a patient who survived for 27 years. On the other hand, 2 weeks was reported as the shortest survival duration after diagnosis [1]. All six patients with radiologically stable disease who were monitored by 3-monthly surveillance imaging in the present study were alive and well at the time of writing this manuscript. These results tend to support the role of surveillance alone for patients with radiologically stable disease, and we believe that patients diagnosed with HEHE should be observed for an initial period of approximately 1–3 months for assessment of the disease biology. If the tumors enlarge rapidly (increase of > 3 cm in 3 months), they should receive appropriate treatment. Moreover, LT should be considered if there is radiological evidence of diffuse or progressive disease. Figure 5 presents a flow diagram showing the sequence of treatment selection for patients with this rare but intriguing disease.

## Conclusions

HEHE is a rare vascular malignancy with nonspecific clinical and laboratory findings. The imaging features can easily lead to misdiagnosis. Diagnosis of HEHE is difficult and primarily depends on the findings of histopathology and immunohistochemistry. Currently used treatment options for HEHE include LT, LR, RFA, chemotherapy, and observation without treatment. On the basis of our findings, we suggest that initial observation may be a key step in the management of HEHE, considering its indolent clinical course. If the lesions progress during the observation period, LR or LT should be scheduled as appropriate. However, our results are limited by the small sample size, and further studies are necessary to gather more information that can aid in appropriate treatment planning for this rare tumor.

## Data Availability

The datasets used or analyzed during the current study are available from the corresponding author on reasonable request.

## Abbreviations

CT: Computed tomography
EHD: Extrahepatic disease
EHE: Epithelioid hemangioendothelioma
HEHE: Hepatic epithelioid hemangioendothelioma
LR: Liver resection
LT: Liver transplantation
MRI: Magnetic resonance imaging
RFA: Radiofrequency ablation
